# Sustained Release Intraocular Drug Delivery Devices for Treatment of Uveitis

**Published:** 2011-10

**Authors:** Nahid Haghjou, Masoud Soheilian, Mohammad Jafar Abdekhodaie

**Affiliations:** 1 Department of Petroleum and Chemical Engineering, Sharif University of Technology, Tehran, Iran; 2 Ophthalmic Research Center, Shahid Beheshti University of Medical Sciences, Tehran, Iran

**Keywords:** Uveitis, Drug Delivery, Sustained-release, Corticosteroid, Steroid-sparing Medications

## Abstract

Corticosteroids have been the mainstay of uveitis therapy. When intraocular inflammation is unresponsive to steroids, or steroid related side effects become a concern, steroid-sparing medications may be administered which can be classified into immunosuppressive and immunomodulatory agents. Uveitis treatment can be delivered systemically, topically, periocularly or intraocularly. All of the above mentioned medications can entail significant systemic side effects, particularly if administered for prolonged durations, which may become treatment-limiting. Some medications, particularly hydrophobic compounds, may poorly cross the blood–retinal barrier. Topical medications, which have the least side effects, do not penetrate well into the posterior segment and are unsuitable for posterior uveitis which is often sight-threatening. Intraocular or periocular injections can deliver relatively high doses of drug to the eye with few or no systemic side effects. However, such injections are associated with significant complications and must often be repeated at regular intervals. Compliance with any form of regular medication can be a problem, particularly if its administration is associated with discomfort or if side effects are unpleasant. To overcome the above-mentioned limitations, an increasing number of sustained-release drug delivery devices using different mechanisms and containing a variety of agents have been developed to treat uveitis. This review discusses various current and future sustained-release ophthalmic drug delivery systems for treatment of uveitis.

## INTRODUCTION

Uveitis is an umbrella term covering a large group of ocular inflammatory disorders that primarily involve the uvea but may also affect adjacent tissues. In intermediate uveitis, the primary focus of inflammation is the vitreous, whereas in posterior uveitis, the retina or choroids are afflicted.^1^ Uveitis may also be categorized as infectious and noninfectious. Intermediate and posterior uveitis may occur as a primary ocular process or can be the manifestation of a systemic disease. They account for much of the visual loss associated with uveitis due to the high rate of complications including cystoid macular edema (CME), subretinal and epiretinal fibrosis, retinal detachment, optic atrophy, glaucoma, and cataracts. A European study involving over 500 patients with posterior uveitis reported that up to 35% suffered from blindness or visual impairment.^2^ Furthermore, 10–15% of blindness in the USA is attributed to uveitis.^3^

The main goal in the treatment of uveitis is to eliminate intraocular inflammation, relieve discomfort and prevent visually significant complications. When anti-inflammatory agents are given systemically, they often need to be administered at high doses over long periods to achieve adequate anti-inflammatory effect. Corticosteroids are the mainstay of uveitis therapy; however, treatment may not be fully effective, or side effects may be treatment-limiting. The side effects of chronic systemic corticosteroid administration have been well documented and include changes in general appearance, weight gain, systemic hypertension, hyperglycemia, gastritis, opportunistic infections, and psychosis. Under such circumstances it is often necessary to switch to alternative drugs. These agents can be broadly termed steroid-sparing drugs since they can either reduce the required dose of corticosteroids or may replace them altogether. Broadly speaking, steroid-sparing medications can be classified into immunosuppressive and immunomodulatory agents. Immunosuppressive agents include antimetabolites, such as methotrexate, azathioprine and mycophenolate mofetil; and alkylating agents, such as cyclophosphamide and chlorambucil. Immunomodulatory agents include calcineurin inhibitors such as cyclosporin A (CsA) and tacrolimus (FK506), and biological agents such as infliximab and adalimumab.

Uveitis treatment can be delivered topically, periocularly, intraocularly or systemically. There are problems common to all delivery routes and some specific to each of them.

All of the previously outlined medications have significant systemic side effects particularly if used for prolonged periods of time which can become treatment-limiting. Many immunosuppressive drugs are teratogenic and contraindicated during pregnancy; some of them may even prevent conception. Most side effects become apparent during treatment and can damage specific organs such as the liver and kidneys, the function of which needs to be continuously monitored during the course of treatment. However, certain side effects such as osteoporosis and lymphoproliferative malignancies may not become apparent until years after treatment has been terminated. Osteoporosis is particularly related to corticosteroid use even at low doses. Rapid bone loss has been associated with corticosteroid doses >5 mg employed for three months or more. Long-term immunosuppression may increase the risk of cancer, particularly solid tumors and lymphomas. This increased cancer risk is probably the result of reduction in normal immune surveillance or due to the direct effect of the medication on DNA.

Compliance with any form of regular medication can be a problem, particularly if its administration is associated with discomfort or if side effects are unpleasant. Some medications, particularly hydrophobic drugs, may cross the blood–retinal barrier poorly, which is an important consideration for all delivery systems except for intraocular injections. Topical medications, which entail the least side effects, do not penetrate into the posterior segment and are unsuitable for posterior uveitis, which is often sight-threatening.

Intraocular or periocular injections can deliver a relatively high dose of drug to the eye with few or no systemic side effects. However, these methods may be associated with significant complications and often need to be repeated at regular intervals. Repeated periocular injections may also lead to complications including globe perforation, orbital fibrosis and ptosis.^4^

To overcome the above mentione d limitations, an increasing number of sustained-release drug delivery devices using different mechanisms and containing a variety of agents have been developed to treat uveitis.

This article reviews major drug delivery technologies currently in clinical trials or at experimental stages for treatment of uveitis.

## SOLID IMPLANTS

### Retisert

In April 2005, Retisert (Bausch & Lomb, Inc., Rochester, NY, USA) received fast-track approval by the US Food and Drug Administration (FDA) as an orphan drug for treatment of chronic noninfectious uveitis affecting the posterior segment. This type of uveitis is extremely damaging because it tends to strike people in their prime working years and unless controlled, can lead to tissue destruction and vision loss. Retisert is a sterile implant that releases the potent anti-inflammatory corticosteroid fluocinolone acetonide (molecular weight, 452.5 Da).

Fluocinolone acetonide is a white crystalline powder, insoluble in water but soluble in methanol. Each Retisert consists of a tablet containing 0.59 mg of the active agent, fluocinolone acetonide, and the following inactive ingredients: microcrystalline cellulose, polyvinyl alcohol, and magnesium stearate. The drug release rate is 0.6μg/day initially, which decreases over the first month to a steady state between 0.3–0.4 μg/day up to approximately 30 months.

**Figure 1:**
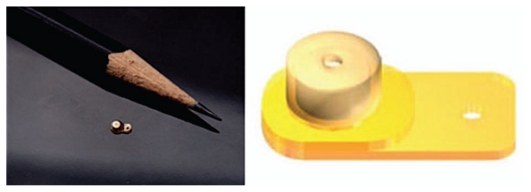
Retisert implant.

Retisert is about the size of a grain of rice and consists of a tablet encased in a silicone elastomer cup containing a release orifice and a polyvinyl alcohol (PVA) membrane positioned between the tablet and the orifice (Fig. 1). The silicone elastomer cup assembly is attached to a PVA suture strut with silicone adhesive. The liquid environment of the vitreous hydrates the pellet and puts some drug in the solution which crosses the implant’s orifice. Retisert has linear release characteristics with no bolus release in the beginning.

Use of a Retisert becomes more of a consideration when there is intermediate or posterior uveitis. First it must be ascertained that the uveitis is noninfectious and not associated with a systemic condition with multi-organ involvement such as sarcoidosis. The device is contraindicated for infectious uveitis such as syphilis, toxoplasmosis, cytomegalovirus retinitis, and candidiasis. It may be indicated for noninfectious etiologies including sarcoidosis, Behcet’s disease, and “white dot” syndromes such as multifocal choroiditis or birdshot chorioretinopathy.

It is recommended that treatment of uveitis patients be initiated with a course of oral steroids, primarily prednisolone. In this way, it can be confirmed that the uveitis is noninfectious. It is much easier to discontinue prednisone as compared to remove a Retisert implant if the uveitis is revealed to be infectious. Retisert is also used off-label for treatment of diabetic macular edema.^5^

This fluocinolone acetonide implant has been studied in 3 multicenter, randomized, prospective, phase III controlled clinical trials. Two of them were double-masked and compared 2 doses (0.59 mg vs. 2.1 mg) of the implant in one eye compared to no treatment in control eyes.^6^,^7^ By continuous delivery of therapy to a localized area, Retisert can reduce the rate of recurrence of uveitis. During the clinical trials leading to FDA approval, the rate of recurrence decreased from 54% before to 7% after Retisert implantation; and from 40% before to 14% after implantation of the device in two different trials. Retisert also stabilized or improved visual acuity in 80% of patients. Retisert reduced the percentage of patients requiring systemic corticosteroid therapy from 47–63% to 5–10% after 34 weeks.

Pavesio et al^8^ performed an open-label trial comparing the 0.59 mg implant vs. standard of care (SOC). It was observed that eyes receiving Retisert experienced delayed onset and a lower rate of recurrence of uveitis as compared to SOC eyes (18.2% vs. 63.5%). Common ocular adverse events in implanted eyes included elevated intraocular pressure (IOP) requiring IOP-lowering surgery (21.2%) and cataracts requiring extraction (87.8% of phakic eyes). The authors concluded that Retisert may be considered a reasonable alternative when patients are intolerant or refractory to systemic or topical therapy. No non-ocular adverse events were observed in the implant group, whereas such events occurred in 25.7% of subjects in the SOC group. Therefore, implantation may be considered for patients in whom systemic steroid-related side effects are more frequent and/or severe than ocular adverse effects.^9^ Patients should be informed about potential adverse effects of the fluocinolone acetonide intravitreal implant including cataracts, increased intraocular pressure or hypotony, endophthalmitis and need for additional procedures. Because of the differing benefits and risks of treatment with intravitreal implants in comparison with systemic therapy, patients should make an informed choice between such treatments. Some patients and insurance companies may be taken aback by Retisert’s $18,000 price tag. But education regarding the even higher cumulative cost of alternative treatments could facilitate acceptance by patients and reimbursement by insurance companies.

The “Multicenter Uveitis Steroid Treatment” (MUST) trial, sponsored by US National Institute of Health, is currently evaluating the effectiveness of Retisert as compared to conventional therapy (oral corticosteroids) for management of posterior uveitis in 400 patients at 20 sites throughout North America. This five-year study is presently recruiting patients.

### Iluvien (Medidur)

**Figure 2:**
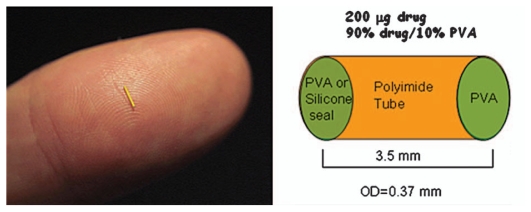
Iluvien implant shown on a human finger to indicate size.

Inflammation in the posterior segment may lead to macular edema and other retinal complications. Iluvien, previously known as Medidur FA (Alimera Sciences, Alpharetta, GA, USA), is an injectable non-erodible intravitreal device for treatment of diabetic macular edema (DME)^10^ which is a common complication of diabetic retinopathy and a leading cause of visual loss in subjects less than age 65. Similar to Retisert, Iluvien also contains fluocinolone acetonide (180 μg). Unlike Retisert however, Iluvien is injected into the eye as an office based procedure using a proprietary inserter with a 25-gauge needle, which allows a self-sealing wound. The method of administration is similar to an intravitreal injection. Iluvien is a tiny cylindrical polyimide tube, 3.5mm in length and 0.37mm in diameter and releases a low dose of 0.23–0.45 μg/day fluocinolone acetonide for 18 to 36 months after injection (Fig.2). Iluvien is non-erodible and remains in the vitreous cavity even after drug release has been exhausted. Therefore, patients requiring repeated injections may end up with multiple devices trapped in the vitreous base for an indefinite period of time.

Iluvien may have a more favourable ocular hypertension side-effect profile as compared to Retisert. This may have to do with the position of these implants relative to the ciliary body and/or the trabecular meshwork.

Two different models of the device releasing high dose (0.45 μg/day) and low dose (0.23 μg/day) active component have been designed for 18 and 36 months of drug release and are currently being investigated in two global phase 3 pivotal clinical trials involving 956 patients which has been granted “fast track” by the FDA.

### Surodex

Surodex (Allergan, Inc., Irvine, CA, USA) is a rod-shaped biodegradable matrix implant 1.0×0.5 mm in size consisting of dexamethasone (molecular weight, 392.47) and poly lactide-co-glycolide acid (PLGA) with hydroxypropyl methylcellulose (HPMC) which provides sustained drug release at a constant rate of 60 μg over 7–10 days.^11^

The implant is inserted into the anterior chamber following cataract surgery to control postoperative inflammation.^12^ Surodex does not require suture fixation and is well tolerated.^13^ In cataract patients, Surodex has been shown to reduce anterior chamber cell and flare in the postoperative period and to have an anti-inflammatory effect at least as good as that of topical steroids.^14^ In another study, Surodex was implanted in the anterior chamber of rat eyes after induction of acute endotoxin induced uveitis (EIU) and chronic experimental autoimmune uveitis (EAU). Both acute and chronic inflammatory reactions were markedly inhibited in eyes receiving Surodex implants.^15^

### Ozurdex (Posurdex)

**Figure 3:**
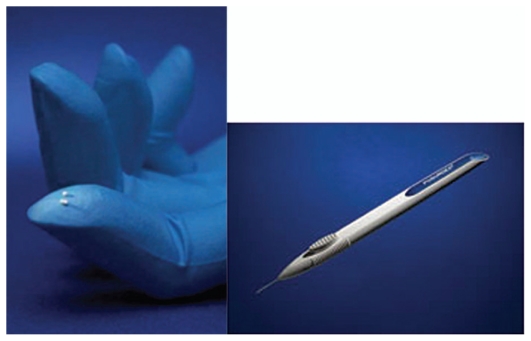
Ozurdex dexamethasone intravitreal implant and its applicator.

Ozurdex is an intravitreal implant containing 0.7 mg dexamethasone in a NOVADUR solid polymer drug delivery system. Ozurdex is preloaded into a single-use, specially designed applicator to facilitate injection of the rod-shaped implant directly into the vitreous cavity (Fig. 3).

**Figure 4:**
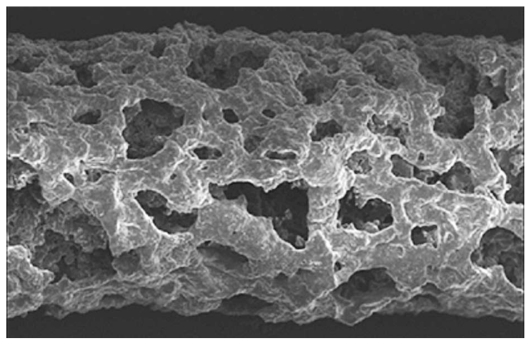
Ozurdex’s biodegradable matrix three weeks after implantation.

The NOVADUR system contains PLGA intravitreal polymer matrix. The PLGA matrix slowly degrades to lactic acid and glycolic acid, meaning that when the active agent is consumed, degradation products are water and carbon dioxide, leaving no residue in the eye (Fig. 4).

Ozurdex is preservative-free and was initially called Posurdex; it has a history dating back almost a decade. Allergan partnered in 2001 with the original developer of the implant, Oculex, and then purchased this company in 2003. Ozurdex was approved for treatment of macular edema following branch or central retinal vein occlusion (RVO) in June 2009, with approval for additional indications anticipated.^16^ Allergan, Inc. has completed a phase 3 trial of Ozurdex for treatment of ocular inflammation in the setting of posterior and intermediate uveitis. The drug is currently under FDA review for this indication. Clinical data for uveitis is promising and once approved, Ozurdex will be a valuable option for uveitis.

The implant is placed via a 22-gauge applicator into the vitreous cavity. It can deliver dexamethasone for up to six months with a relatively mild side effect profile.^17^ Some increase in IOP, usually peaking around day 60, has been observed which returns to baseline by month six. Maximum effectiveness, as measured in pivotal clinical trials, occurs between 60 and 90 days.

Ozurdex is particularly effective in vitrectomized eyes, where traditional intravitreal injections clear too quickly to be of much use.

### I-vation

Based on experimental studies performed by Machemer, Peyman and others, as well as clinical observations, intravitreal injections of triamcinolone acetonide (TA) have increasingly been used for treatment of uveitis. TA (molecular weight, 434.5 Da) is designated chemically as 9-fluoro-11b,16a,17,21-tetrahydroxypregna-1, 4-diene-3,20-dione cyclic 16,17-acetal with acetone. The empirical formula is C_24_H_31_F_O6_.

In order to achieve sustained therapeutic levels of TA and to reduce the frequency of intravitreal injections, different implantable devices or injectable systems have been under investigation. However no sustained-release TA device is yet commercially available.^18^

**Figure 5:**
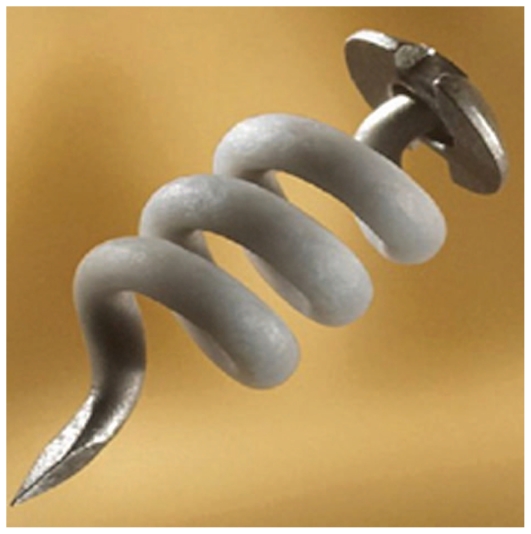
I-vation sustained drug delivery system.

SurModics Inc. has designed a delivery system using I-vation technology capable of delivering TA on a sustained-release basis. The I-vation intravitreal TA implant consists of three components: a helix-shaped non-ferrous metallic scaffold, a cap attached to the helix, and a drug-loaded polymer coating that encapsulates the helix (Fig. 5). The polymers are polymethylmethacrylate and poly- ethylene-co-vinyl acetate. The helical shape of the scaffold maximizes the surface area available for drug coating and enables suture-less anchoring of the implant against the sclera. The thin cap is designed to reside under the conjunctiva. Insertion of the implant requires conjunctival cut-down and placement in an operating room through a 0.5-mm needle stick. The delivery system is non-biodegradable and remains in place after drug release is completed.

The I-vation platform offers a great deal of versatility and flexibility for formulation and pharmacokinetic control. The device is implanted through a 25-gauge needle stick and is self-anchoring within the sclera. I-vation is an example illustrating the risks associated with the development of sustained-release devices. I-vation was designed to deliver 925μg TA over a period of up to two years. A phase 1 trial including 30 patients showed reduction in DME at 24 months. However, when study data released in 2008 favored focal/ grid photocoagulation over preservative-free intravitreal TA for treatment of DME, the phase 2b I-vation trial was suspended.^19^

### Vitrasert

**Figure 6:**
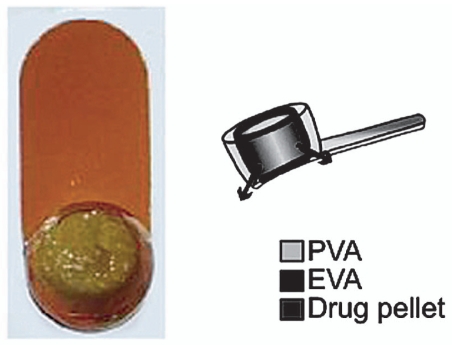
Front view of Vitrasert on the left and the schematic on the right.

The Vitrasert reservoir-type device, currently marketed by Bausch+Lomb, was developed by Paul Ashton, P. Andrew Pearson, and Thomas Smith at the University of Kentucky. It is the first implantable ganciclovir delivery device approved by the FDA in 1996 for treatment of cytomegalovirus CMV retinitis^20^ (Fig. 6). The device is composed of drug and polymeric coats of polyvinyl alcohol (PVA) and ethylene vinyl acetate (EVA). PVA, a permeable polymer, regulates the rate of ganciclovir release and EVA, an impermeable polymer, limits the surface area of the device through which the active agent can be released. The device has shown to have no initial burst effect. Currently, the same type of implant containing dexamethasone^21^, fluocinolone acetonide^22^, or cyclosporine^23^ is being tested for treatment of severe uveitis.

The commercially available device is relatively large; it requires a 4–5mm sclerotomy at the pars plana for implantation and releases the drug for 5 to 8 months. Since it is nonbiodegradable, the drug-depleted device needs to be removed during a second procedure in order to implant another device if required.

### Cyclosporine Devices

While systemic and local corticosteroids are often effective in treating uveitis, an alternative treatment may be required in eyes with a history of steroid-induced glaucoma or those refractory to steroid therapy.

Cyclosporin A (CsA) is a naturally occurring hydrophobic macrolide produced by soil fungi and a potent immunosuppressive agent that selectively suppresses T-cell activation by inhibiting the phosphatase action of calcineurin, thereby suppressing transcription of interleukin 2 and other early phase T-cell activation genes.^24^ Such inhibition prevents clonal expansion of helper and cytotoxic T-cells. CsA also prevents chemotaxis of inflammatory cells, particularly that of eosinophils. Peak CsA blood levels are reached six hours after ingestion; the drug is metabolized and concentrated by the cytochrome P450 microsomal system in the liver.

CsA penetrates the eye poorly when administered topically.^25^ Accordingly, topical CsA is not generally administered for control of intraocular inflammation. In contrast, moderate intraocular CsA levels are achieved with oral and systemic administration.^26^

Side effects of systemic CsA may include oral ulceration and gingivitis, hypertrichosis, malaise, headaches, muscle cramps, and gastrointestinal disturbance. Serious side effects such as nephrotoxicity and hypertension may be treatment-limiting.^27^

Prolonged use of cyclosporine, and thus good patient compliance, is required to adequately control intraocular inflammation. Direct intraocular injection of cyclosporine has been shown to control intraocular inflammation in an animal model of uveitis.^28^ However, the half-life of intravitreal cyclosporine is short which limits its effectiveness in chronic forms of uveitis.^29^

Gilger and coworkers^30^ described a discoid intravitreal device developed for the constant release of cyclosporine (CsA) in inflammatory episodes of uveitis in horses. This was accomplished by monitoring clinical signs and intraocular damage; and measuring cellular infiltrates, T-lymphocyte counts and transcribed cytokine specific mRNA. Nine healthy horses were immunized peripherally with H37RA-mTB antigen twice, and then received 25mg of H37RA-mTB antigen intravitreally in the right eye and an equal volume of balanced salt solution in the left eye. Two weeks later, the animals randomly received either the CsA-loaded device or a placebo in both eyes. One week after implantation of the devices, 25mg of H37RA-mTB antigen was re-injected into the right eye of each animal. Clinical signs of ophthalmic inflammation were graded following injections and implantation. Aqueous and vitreous protein levels, infiltrating cell counts, total number of T-lymphocytes, and levels of IL-2 and IFNG-mRNA were significantly less in eyes containing the CsA device as compared to eyes with placebo. CsA devices did not completely eliminate recurrent experimental inflammatory episodes in the horse model; however, the duration and severity of inflammation, cellular infiltration, tissue destruction, and levels of pro-inflammatory cytokine RNA transcripts were significantly less in eyes implanted with the CsA devices. This study was focused on demonstrating the effectiveness of delivering CsA via an intraocular device for treatment of immune-mediated intraocular inflammation in an equine eye model. The flexibility of the device for delivering other drug molecules and its efficacy in other animal models or human subjects is yet to be reported.

In another study, glycolide-co-lactide-co-caprolactone copolymer (PGLC) implants loaded with 2 mg CsA were tested in a rabbit model of uveitis. Eyes receiving these implants showed constant drug release for at least 14 weeks along with reduction of intraocular inflammatory processes.^31^

## INJECTABLE SYSTEMS

### Verisome Delivery System

**Figure 7:**
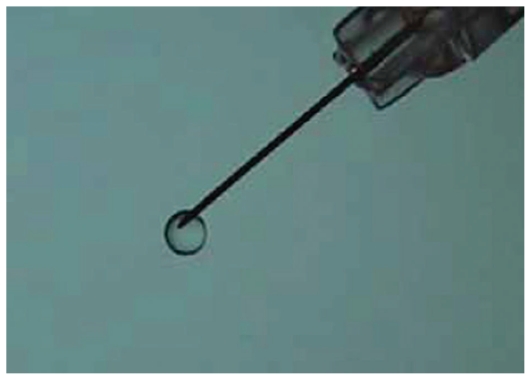
Verisome delivery system.

Icon Biosciences Inc. (Sunnyvale, CA, USA) is developing a promising drug delivery technology called Verisome. According to its manufacturer, this system can be used to release a broad range of pharmaceutical agents including small molecules, peptides, proteins, and monoclonal antibodies. The basic technology is highly versatile and can be formulated into numerous products, as a biodegradable solid, gel, or liquid substance that provide drug release in a controlled manner over some weeks to a year for ocular, systemic, or topical applications.^32^ Ophthalmic applications are focused on the ability of this system to create an injectable liquid or slightly viscous gel. Verisome-based products can be injected into the vitreous as a liquid via a standard 30-gauge needle (Fig. 7). When the drug is injected into the vitreous, it coalesces into a single spherule that settles inferiorly. The system is biodegradable and versatile for administering different drugs; furthermore duration of use can be tailored. Shrinkage of the Verisome spherule over time reflects simultaneous degradation of the delivery system and release of the active agent. In ophthalmology, this mode of delivery offers advantages because the physician can easily assess the status of therapy by observing the drug-containing system within the eye. When the spherule is no longer visible, the entire drug has been released, and no vehicle remains in the eye. In the future, flexibility of the system, along with the ability to directly monitor its status, may allow physicians to individually tailor the duration of drug delivery, potentially leading to cost efficiency and better results. Rather than having therapy dictated by the design of the delivery vehicle, physicians may be able to administer drugs with what they deem to be the appropriate duration and intensity of treatment for each patient.

**Figure 8:**
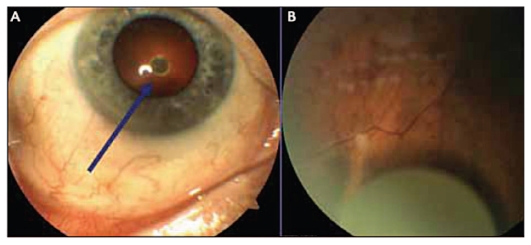
Slit lamp photograph of the Verisome immediately after injection before it has settled down in the vitreous (A). Fundus photograph of the Verisome inside the eye, sitting below the visual axis (B).

In its first clinical trial, IBI 20089, the Verisome technology was formulated for delivery of TA.^33^ The injected gel forms a spherule in the posterior chamber after injection (Fig. 8) which gradually degrades and disappears as the drug is released. This liquid-gel formulation is designed to last up to 1 year, with duration determined by the volume injected. Follow-up visits to assess safety and efficacy of the drug delivery system included visual acuity and IOP measurements, optical coherence tomography, fundus photography and biomicroscopy. In the phase 1 study, Verisome was well tolerated and showed efficacy in patients with CME secondary to RVO. No injection-related adverse events or safety concerns were noted during the trial. Controlled-release biologic efficacy was also observed on sequential OCTs over 1 year. A phase 2–3 pivotal clinical study is anticipated to begin soon.

### Cortiject Implant

The Cortiject implant (NOVA63035, Novagali Pharma) is a preservative- and solvent-free injectable emulsion based on the EYEJECT technology platform that contains a tissue-activated corticosteroid prodrug. The prodrug is converted into the active agent by enzymes present in the target tissues, i.e. retina and choroid. These enzymes are not present in the vitreous or in the aqueous humor. The specific distribution of enzymes may avoid common corticosteroid related side effects such as glaucoma and cataract.

A single intravitreal injection of the emulsion provides sustained release of corticosteroid over a 6 to 9 month period. An open-label, phase 1, dose-escalation clinical study to assess the safety and tolerability of NOVA63035 in patients with DME is currently under way. The patients will be monitored for 12 months following injection.

### Particulate Drug Delivery Systems

Particulates are commonly classified into microparticles and nanoparticles based on size. Nanoparticles are colloidal particles ranging from 10 to 1,000 nm, in which drug may be entrapped, encapsulated, and/or absorbed. Microparticles are small drug-containing polymeric particles 1–10μm in size, which are suspended in a liquid carrier medium.

Microcapsules are spherical entities in which drug particles or droplets are entrapped in a polymeric membrane. Microspheres are a polymer–drug combination in which the drug is homogeneously dispersed within the polymeric matrix. Nanoparticles are divided into nanospheres where the drug is either incorporated within or attached to the surface, and nanocapsules that have a central cavity surrounded by a polymeric membrane.^34^ To avoid opsonisation and recognition by host phagocytes, the surface of the particles can be modified by pegylation.

De Kozak et al^35^ used polyethylene glycol-coated cyanoacrylate nanoparticles loaded with tamoxifen for inhibition of intraocular inflammation in a rat model of EAU. These nanoparticles significantly inhibited the severity and extent of uveitis in treated eyes without any detectable ocular toxicity.

In-vivo studies on nanoparticles of piroxicam (a non-steroidal anti-inflammatory drug) using the solvent evaporation/extraction technique, and of methylprednisolone acetate (MPA) formulated using a copolymer of poly (ethylacrylate, methyl-methacrylate and chlorotrimethylammonioethyl-methacrylate) revealed that inflammation was inhibited by nanoparticles suspension more efficiently than microsuspension of the drug alone in rabbit eyes with EIU.^36^

Herrero-Vanrell and Refojo^37^ developed PLGA microspheres for sustained delivery of dexamethasone to prevent uveitis following surgical procedures. In their study, the inherent viscosity of PLGA (50% lactide-50% glycolide) was 0.2 dL/g and the proportion of dexamethasone in the polymer was 2/10. Ten milligrams of the microspheres contained 1410μg of the active agent that was released in vitro for at least 45 days. The intravitreal half-life of dexamethasone administered via direct intravitreal injection is about 3.48 hours.^38^

### Liposomes

During the last decade, the use of liposomes as a delivery system has become an attractive subject of research.^39^ Liposomes are small vesicles, typically ranging in size from 0.01 to 10 mm, composed of a single phospholipid layer or concentric bilayers entrapping water in their center. They are formed by the dispersion of phospholipids in water and have been investigated since the 1970s as a means to achieve controlled and targeted drug delivery. Hydrophilic drugs can be entrapped within the aqueous core while lipophilic drugs may be incorporated into the lipid bilayers. Both of these integration processes can dramatically alter the biodistribution of liposomes.^40^

One study demonstrated the potential use of liposomes for intraocular drug delivery.^41^ The authors evaluated the use of vasoactive intestinal peptide (VIP) for treatment of intravitreal inflammation. This neuropeptide interacts with many cell types associated with inflammation, including endothelial cells, macrophages and lymphocytes. In vitro, VIP inhibits the secretion of chemokines, nitric oxide and inflammatory cytokines. Additionally, it enhances the production of the anti-inflammatory cytokine IL-10. When bonded to liposomes, VIP has been used to treat arteritis and several other inflammatory conditions in experimental models.

In another study, rats with EIU were treated with an intravitreal injection of saline, saline containing VIP, liposomes containing VIP or unloaded liposomes.^42^ Twenty-four hours after treatment, VIP concentration in ocular fluids was 15 times higher in eyes injected with liposomal VIP compared to the saline/VIP preparation. In VIP-liposome injected eyes, macrophages, and inflammatory cytokine and chemokine mRNA expression were significantly reduced as compared to the eyes injected with saline, saline-containing VIP or unloaded liposomes.

In another study, the authors investigated the effects of adding hyaluronic acid to a VIP liposomal delivery system.^43^ This study showed significant reduction in inflammation and suggested that the addition of hyaluronic acid further enhanced the efficacy and duration of effect of liposomal VIP. The prospect of an ocular anti-inflammatory medication that is not only effective but also lacks the conventional side effects of steroids is very encouraging.

## TRANSSCERAL IONTOPHORESIS

Iontophoresis is an active method of drug delivery which employs a small electrical current to enhance diffusion of drug molecules across an intact sclera.^44^ As a non-invasive and well-tolerated method, iontophoresis has the potential to replace systemic administration and repeated intravitreal injections for posterior eye diseases.^45^ Eyegate (Optis Group, Paris, France) and OcuPhor (IOMED, Salt Lake City, USA) are two main ophthalmic iontophoresis systems under investigation.

**Figure 9:**
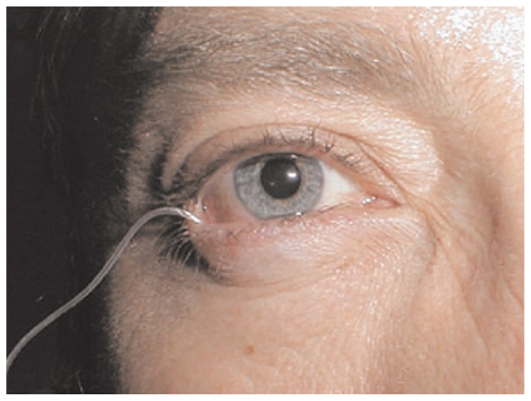
OcuPhor iontophoresis system inserted in the human eye. Applicator is placed on the sclera under the lower eyelid.

The OcuPhor system consists of a drug applicator, a dispersive electrode, and an electronic iontophoresis dose controller. The drug applicator is a small silicone shell that contains a patented silver-silver chloride ink conductive element, a hydrogel pad to absorb the drug formulation, and a small flexible wire to connect the conductive element to the dose controller. The drug pad is hydrated with drug solution immediately prior to use, and the applicator is placed on the sclera under the lower eyelid (Fig. 9).

**Figure 10:**
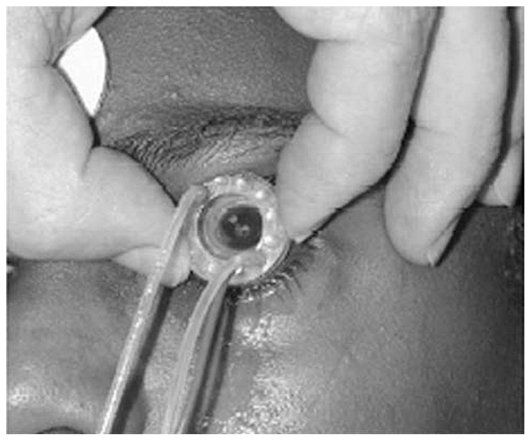
Eyegate iontophoresis system applied on the human eye.

Eyegate consists of an annular silicone probe for transscleral iontophoresis with 0.5 cm^2^ contact area and a 13 mm opening to avoid contact with the cornea (Fig. 10).

Iontophoretic delivery of anti-inflammatory drugs into the eye has been studied in humans and various animal models, and offers a viable alternative to topical or systemic administration.^46^

Lachaud^47^ used iontophoresis for delivery of hydrocortisone acetate (0.1% solution) into rabbit eyes using a current of 3 milliamperes (mA) for 10 minutes. The author demonstrated that iontophoresis could deliver higher concentrations of steroid into rabbit eyes than topical (0.5%), or subconjunctival (0.1mL, 2.5%) routes. In human studies, Lachaud used iontophoresis to deliver dexamethasone acetate (7 mg, 1–2mA, 20 min) and treat a variety of clinical conditions, including idiopathic uveitis. The author reported that a significant proportion of patients with uveitis experienced more rapid recovery and/or increased comfort. Lachaud concluded that iontophoresis achieved therapeutic concentrations of the steroid(s) in ocular tissues. However, this open clinical study did not involve comparison with eyes receiving other therapies or with untreated control eyes.

Lam et al^48^ delivered 30% dexamethasone solution by transscleral iontophoresis into rabbit eyes using 1.6mA for 25 minutes. The diameter of the cylinder holding the drug solution in contact with the sclera was 0.7 mm. They compared peak steroid concentrations in the choroid and retina following iontophoresis, subconjunctival injection (1 mg) or retrobulbar injection (1 mg). Peak steroid concentration (mg/g tissue) was 122 for iontophoresis, 18.1 for subconjunctival injection, and 6.6 for retrobulbar injection. In the vitreous humor, corresponding values were 140, 0.2, and 0.3 mg/mL, respectively. Even 24 hours after iontophoresis, significant therapeutic levels of dexamethasone remained in the vitreous (3.3 mg/mL) and in the choroid-retina (3.9 mg/g).

Eljarrat-Binstock et al^49^ achieved therapeutic dexamethasone levels in different eye segments using a lower current density (5.1mA/cm^2^) for only 4 minutes.

The efficacy of dexamethasone iontophoresis was studied in rat and rabbit models of EIU by Behar-Cohen et al^50^ and Hastings et al.^51^ The first authors used a 6 mm diameter eye cup covering the cornea and sclera, while the latter investigators used a saturated hydrogel applicator in the superior cul-de-sac. The applied electrical current was 0.4mA (1.2 mA/cm^2^) for 4 minutes by Behar-Cohen et al, and 4mA for 20 minutes by Hastings et al. Both studies showed that administration of dexamethasone by iontophoresis inhibited anterior and posterior segment signs of intraocular inflammation as effectively as systemic administration. Cytokine (TNF-α) expression was inhibited in the anterior as well as the posterior segment. No clinical or histological damage was caused by iontophoresis. Thus, iontophoresis can deliver therapeutic doses of anti-inflammatory drugs to the posterior as well as the anterior segment of the eye and may be a viable alternative to systemic administration of steroids in severe ocular inflammation.

Recently, EyeGate Pharma announced the completion of a phase II study of EGP-437 (dexamethasone phosphate ophthalmic solution optimized for iontophoresis) delivery using iontophoresis technology for treatment of anterior uveitis. The company submitted the anterior uveitis study results and data from a completed phase II study in dry eye patients to the FDA as part of an end-of-phase II meeting.

The EGP-437 phase II data is encouraging because it not only shows promising signs of efficacy but addresses compliance issues, by providing direct control of dosing. These positive results demonstrate that iontophoretically delivered drugs may offer new treatment options.

## CONCLUSIONS

Uveitis is a potentially sight-threatening disease which has traditionally been treated with topical corticosteroids supplemented by periocular, intravitreal, and systemic steroids in severe cases. Chronic use of local and systemic corticosteroids can result in significant side effects that are sometimes life-threatening. Although newer immunosuppressive agents can reduce the amount of systemic steroids required for control of sight-threatening uveitis, most of these agents are poorly absorbed by the eye when applied topically and entail significant side effects. The chronic nature of uveitis also requires long-term patient compliance with prescribed medications. Sustained-release devices used for uveitis allow local delivery of immunosuppressive agents in adequate concentrations without systemic side effects. To date, for selected patients, potential complications associated with the insertion of these devices do not outweigh their advantages. However, miniaturization of the implants, allowing direct injection without need for complicated surgery, is a necessary development avenue. Particulate systems which can be engineered to target specific cells or tissues are another promising alternative.

A range of anti-inflammatory medications, some of which have been used systemically for uveitis and some have not, may be used in sustained-release devices in the future. Designing non-invasive sustained drug delivery systems and exploring the feasibility of topical application to deliver drugs to the posterior segment may drastically improve drug delivery in coming years. Future horizons could include devices containing complementary drugs and responsive sustained release delivery systems.
